# Psychopathological factors and personality dimensions on dysfunctional eating behaviors in a sample of individuals with obesity

**DOI:** 10.3389/fpsyg.2023.1140890

**Published:** 2023-09-19

**Authors:** Margherita Attanasio, Antonio Giuliani, Lucia Romano, Cristina Laidò, Gilda Di Poggiovalle, Ilenia Le Donne, Valentina Di Fonzo, Sergio Tiberti, Marco Valenti, Monica Mazza

**Affiliations:** ^1^Department of Biotechnological and Applied Clinical Sciences, University of L’Aquila, L’Aquila, Italy; ^2^Department of Surgery, San Salvatore Hospital, L'Aquila, Italy; ^3^Istituto Romano di psicoterapia e psicodinamica integrata, Rome, Italy

**Keywords:** obesity, eating disorders, dysfunctional eating behaviors, bariatric surgery, mediation analysis, psychological evaluation

## Abstract

**Introduction:**

Obesity and eating disorders are considered to be part of a broad spectrum of disorders associated with weight issues and maladaptive eating styles. Several studies show that psychopathological and personality characteristics contribute to the development and maintenance of obesity and dysfunctional eating behaviors, showing a bidirectional relationship. The purpose of this study was to understand the role of psychopathological factors and personality dimensions on dysfunctional eating behaviors in a sample of individuals with obesity.

**Methods:**

The study was conducted with 96 individuals with obesity (31 males and 65 females) who underwent psychological assessment. The instruments administered included the Cognitive Behavioral Assessment 2.0 - Primary Scales, the Minnesota Multiphasic Personality Inventory-2, and the Eating Disorder Inventory. Relationships between dysfunctional eating behaviors and personality and psychopathological factors were explored through mediation analysis.

**Results and discussion:**

Our results showed that difficulties related to impulse control and behavior, along with negative and dysphoric emotions, may be associated with difficulties in eating behavior. Mediation analysis showed that the combination of depressive and obsessive-compulsive symptomatology may exacerbate or contribute to the occurrence of eating disorders. These psychopathological aspects should be taken into account during the assessment of patients who decide to undergo bariatric surgery and should be targets of specific psychological interventions.

## 1. Introduction

Obesity is a complex disease with a multifactorial etiology, involving biological, genetic, metabolic, environmental, social, behavioral and psychological aspects (Barbuti et al., 2022; Plasonja et al., 2022). Over the past 50 years, there has been a rapid and progressive global increase in the rate of obesity and overweight, especially in Western and high-income countries (Robinson et al., 2020; Plasonja et al., 2022). Obesity is classified by body mass index (BMI), calculated as the ratio of body weight to the square of the height. Specifically, an adult with a BMI greater than or equal to 30 falls within the obesity range, while a BMI between 25.0 and 29.9 is considered overweight (Lin and Li, 2021). From a clinical perspective, the outcomes of obesity treatments remain largely unsatisfactory due to the high rates of nonresponse and relapse. Literature suggests a bidirectional relationship between obesity and psychopathology (Weiss et al., 2020), and several studies demonstrate that psychosocial and psychopathological factors contribute to the development and maintenance of obesity (Weiss et al., 2020; Asch et al., 2022). Individuals with obesity and overweight are more likely to report low self-esteem, body image disturbance, lowered quality of life, food addiction, stress-related conditions, mood disorders (Chu et al., 2019; Vasiliu, 2022), and impairment in emotional processing (Steward et al., 2016; Scarpina et al., 2021). Recent literature highlights the presence of significant comorbidity between obesity and eating disorders (ED), particularly with binge eating disorder (BED), night eating syndrome and bulimia nervosa, regardless of age or gender (da Luz et al., 2018; Chu et al., 2019; Weiss et al., 2020). ED and obesity are considered two poles of the same continuum and both disorders are associated with maladaptive eating styles that may contribute to their development and maintenance (Segura-Serralta et al., 2020). Patients with both obesity and ED report more weight-related pathology, as well as a higher prevalence of general psychopathology than patients with obesity and without ED (Claes et al., 2013; Guerrini Usubini et al., 2022). Several studies have examined individual differences in psychological factors and personality traits as potential risk factors that may explain why some individuals are unable to engage in health-promoting behaviors (Miller et al., 2006; Mazza et al., 2012; Valenti et al., 2013; De Berardis et al., 2015; Robinson et al., 2020; Vasiliu, 2022). For example, cognitive processes such as inhibitory control may play a key role in impulse control and subsequent reduction in hedonistic eating (Gerdan and Kurt, 2020; Robinson et al., 2020). Individuals with obesity and an ED appear to be less endowed with self-directedness to self-control than individuals with obesity and without an ED (Dalle Grave et al., 2018). Problematic eating behaviors and ED are frequently reported among individuals undergoing bariatric surgery (e.g., sleeve gastrectomy and gastric bypass), a treatment that is currently considered the gold standard for severe obesity (Brode and Mitchell, 2019; Lai et al., 2021; Caltabiano, 2022), especially in cases where there is a failure of conservative weight loss therapies (e.g., lifestyle changes and pharmacological therapy). Studies that have analyzed psychological factors in bariatric patients have found higher levels of general psychopathology, distress, psychosocial dysfunction, obsessive symptoms, impulsivity, neuroticism, lower conscientiousness, a tendency toward introversion, and more emotionally-focused coping (Marek et al., 2017a; Generali and De Panfilis, 2018; Caltabiano, 2022). Literature suggests the importance of considering psychological and personality variabilities in pre-surgery evaluations, as these aspects could significantly influence post-surgery outcomes (Federico et al., 2019; Lai et al., 2021; Barbuti et al., 2022). Based on these assumptions, the purpose of our study is to understand which psychopathological and personality characteristics are associated with dysfunctional eating behaviors in a sample of individuals with obesity who require psychological evaluation to decide whether to undergo bariatric surgery and individuals who, after having surgery, have regained weight. Specifically, our study aims to build a model of the psychopathological characteristics that are most commonly associated with obesity and dysfunctional eating behaviors, which should represent the specific target of psychological interventions.

## 2. Materials and methods

### 2.1. Participants

Participants were 96 individuals with obesity (31 males, 65 females, mean age 44.33 ± 12.51) who consecutively referred to the Laboratory of Clinical Neuropsychology of the Department of Biotechnological and Applied Clinical Sciences, University of L’Aquila, Italy. The inclusion criteria for the recruitment were age ≥ 18 years old, BMI ≥ 30 kg/m^2^, willingness to undergo bariatric surgery, and/or weight regain after surgery. Details about the sociodemographic information of participants are reported in Table 1. The Ethics Committee approved the protocol (prot. n. 18/2019) prior to the recruitment of participants. All participants provided informed consent in accordance with the Declaration of Helsinki. Each participant was assessed individually in a quiet room without any distractions and completed the measures in a single session. A psychologist was present in the room during the administration to provide any further information if necessary.

**Table 1:** Sociodemographic information of participants.

Variable	Male	Female	Statistic test	*p*
*N*	31	65		
Age (years), mean (SD)	46.71 (13.93)	43.51 (12.03)	*t* (94)= 1.16	0.25
Education (years), mean (SD)	11.75 (3,42)	11.63 (3.27)	*t* (94)= 0.17	0.86
Weight (Kg), mean (SD)	140.76 (26.70)	113.02 (17.23)	*t* (94)= 6.13	0.00
Height (cm), mean (SD)	177.24 (8.15)	163.73 (6.81)	*t* (94)= 8.52	0.00
BMI, mean (SD)	42.06 (4.77)	44.64 (6.87)	*t* (94)=1.88	0.06
**Obesity class (%)**				
Class I	6.5	4.6	χ2(2)=2.36	0.31
Class II	16.1	30.8		
Class III	77.4	64.6		

### 2.2. Measures

*Cognitive Behavioral Assessment 2.0 - Primary Scale* (CBA 2.0; Sanavio, 1997) is a battery of ten schedules useful for psychological assessment. It allows the evaluation of several areas including:

- The State–Trait Anxiety Inventory (STAI-X1; STAI-X1/R; STAI-X2; Spielberger et al., 1970), measures used to assess state and trait anxiety;
- Eysenck Personality Questionnaire short form (EPQ-R; Eysenck and Eysenck, 1975) which contains 48 dichotomous items for the evaluation of personality dimensions, such as introversion-extraversion, emotional lability or neuroticism (N), Antisociality and maladjustment (P) and Simulation (L);
- Psychophysiological Questionnaire short form (QPF-R; Pancheri et al., 1986) provides an assessment of stress and psychophysiological disorders;
- Phobias Inventory short form (IP), assesses the subject’s fears;
- The Depression Questionnaire (QD) consisting of 24 items for depressive symptoms;
- Maudsley Obsessive-Compulsive Questionnaire short form (MOCQ-R; Hodgson and Rachman, 1977) which consists of 21 items evaluating obsessive-compulsive symptoms.

The CBA 2.0 also includes an autobiographical and anamnestic schedule (Schedule 4) that investigates the individuals’ personal history and problems, including such aspects as education, work, emotional and sexual relationships, sleep habits, diet, alcohol or drug use, psychological problems, general health status, hobbies, etc.

*Minnesota Multiphasic Personality Inventory-2* (MMPI-2; Butcher et al., 1989) is a personality questionnaire used for the evaluation of personality profile and psychopathology. The questionnaire consists of 567 items with dual response alternatives (“true” or “false”). MMPI-2 includes 10 clinical scales that assess the most significant dimensions of personality, namely Hypochondriasis (Hs), Depression (D), Hysteria (Hy), Psychopathic Deviate (Pd), Masculinity-Femininity (Mf), Paranoia (Pa) Psychasthenia (Pt), Schizophrenia (Sc), Hypomania (Ma), and Social Introversion (Si). The questionnaire also includes 8 validity scales (e.g., indices of validity useful in assessing the acceptability of the protocol), 15 content scales (useful in providing additional information about specific symptoms and describing different personality variables), and 15 Supplemental scales (allow for a more accurate assessment of any clinical problems). Scale scores were calculated using standardized T scores, and a score of >65 indicates the presence of significant psychological problems (Hathaway et al., 1995).

*Eating Disorder Inventory* (EDI; Garner et al., 1983) consists of 64 items that are grouped into 8 subscales that explore, the first 3, attitudes and behaviors related to weight, eating and body appearance, the remaining 5, more general psychological traits. The 8 dimensions assessed by EDI are: drive for thinness (excessive preoccupation with diet, desire to lose weight, and fear of gaining it), bulimia (tendency to binge eating and purging), body dissatisfaction (conviction that some body parts are too big/fat), ineffectiveness (feelings of inadequacy, insecurity, worthlessness and having no control over their lives), perfectionism (excessive personal expectations, drive for success), interpersonal distrust (sense of alienation and reluctance to establish close interpersonal relationships), interoceptive awareness (difficulty in recognizing and identifying emotions and sensations related to hunger and satiety), and maturity fears (the fear of facing the demands of adult life). Using a six-point scale, the subject is asked to indicate how often he or she experiences a particular behavior or symptom from “always” to “never.” Scores are calculated using a 0–3 scale and higher scores indicate more severe symptoms.

### 2.3. Statistical analysis

Using a one sample *z-*test we compared our sample with a sample representative of general population (Adami et al., 1994) on the EDI subscales. In analyzing the results of group differences a Bonferroni correction was applied to α = 0.05 considering the number of comparisons performed, thus, we considered significant *p*-values lower than 0.006. Exploratory Pearson’s correlations were conducted in order to assess relationships between the personality dimensions, assessed with MMPI-2, and dysfunctional eating behaviors, assessed with EDI. Pearson’s correlation analyses were also conducted to assess the associations between psychological constructs, assessed with CBA 2.0, and dysfunctional eating behaviors. Thereafter we performed a stepwise linear regression analysis to identify among personality dimensions those potentially predictors of dysfunctional eating behaviors. A second stepwise regression analysis was conducted to identify which psychological constructs predict dysfunctional eating behaviors. Finally, we created a mediation model on the basis of the regression results. All analyses were performed by using Jamovi Project (2020).

## 3. Results

Mean scores, standard deviations and results of one sample *z-*test for each EDI subscale are listed in Table 2. Our sample compared with the general population sample (Adami et al., 1994) showed significantly higher values on seven EDI scores: Drive for Thinness, Body Dissatisfaction, Bulimia, Interoceptive Awareness, Ineffectiveness, Maturity Fears and Perfectionism.

**Table 2:** Means, SDs, and one-sample *z* statistics for the sample compared with the general population.

	Mean (SD)	*z*	*p*
Drive for thinness	8.7 (5.3)	8.25	<.001^*^
Body dissatisfaction	18.6 (6.5)	13.11	<.001^*^
Bulimia	5.0 (4.9)	4.75	<.001^*^
Interoceptive awareness	5.7 (5.8)	6.69	<.001^*^
Ineffectiveness	5.0 (5.7)	6.45	<.001^*^
Maturity Fears	7.9 (4.8)	7.72	<.001^*^
Perfectionism	4.5 (3.7)	2.97	.002^*^
Interpersonal distrust	4.6 (4.2)	2.21	.007

^*^ significant at *p* < 0.006 according to Bonferroni’s correction.

Table 3 reports the percentages of participants in this study who fall into three categories on T scores of the clinical scales and content scales of the MMPI-2: T scores at or above the cutoff of 65 indicate the presence of psychopathology, T scores between 60 and 64 indicate possible personality tendencies, and scores below 60 fall within the normal range (Pancheri and Sirigatti, 2002; Incerti et al., 2017).

**Table 3:** Percentages of MMPI-2 scales *T*-scores that are ≥ 65, 60–64, and <60.

	*T-Score*		
	≥ 65	60–64	<60
**Clinical scales**			
Hypochondriasis (Hs)	54.2	12.5	33.3
Depression (D)	19.8	9.4	70.8
Hysteria (Hy)	20.8	13.5	65.6
Psychopathic deviate (Pd)	18.8	10.4	70.8
Masculinity – femininity (Mf)	4.2	9.4	86.5
Paranoia (Pa)	9.4	12.5	78.1
Psychasthenia (Pt)	9.4	6.2	84.4
Schizophrenia (Sc)	12.5	7.3	80.2
Hypomania (Ma)	10.4	4.2	85.4
Social introversion (Si)	6.3	7.3	86.4
**Content scales**			
Anxiety (ANX)	10.4	12.5	77.1
Fears (FRS)	14.6	12.5	72.9
Obsessiveness (OBS)	9.4	6.2	84.4
Depression (DEP)	10.4	9.4	80.2
Health concerns (HEA)	36.5	19.8	43.7
Bizarre mentation (BIZ)	7.3	13.5	79.2
Anger (ANG)	6.2	9.4	84.4
Cynicism (CYN)	15.6	16.7	67.7
Antisocial practices (ASP)	9.4	7.3	83.3
Type a behavior (TPA)	9.4	12.5	78.1
Low self esteem (LSE)	6.3	0	93.7
Social discomfort (SOD)	10.4	6.3	83.3
Family problems (FAM)	7.3	7.3	85.4
Work interference (WRK)	10.4	4.2	85.4
Negative treatment indicators (TRT)	10.4	12.5	77.1

Table 4 shows the percentages of participants reporting clinically significant scores on the CBA 2.0. Raw scores were transformed into percentiles based on comparisons with Italian normative data (Sanavio, 1997). Three percentile ranges of distribution were considered for this study (<25°, 25°–75°, >75°).

**Table 4:** CBA 2.0, percentages of participants at three cut-off (<25°, 25°–75°; >75°).

Schedule	<25°	25°-75°	>75°
State anxiety (STAI-X1)	40.6	39.6	19.8
Trait anxiety (STAI-X2)	47.9	42.7	9.4
Intra/extroversion (EPQ/R-E)	15.6	43.8	40.6
Emotional lability or neuroticism (EPQ/R-N)	54.1	38.5	7.3
Antisociality and maladjustment (EPQ/R-P)	46.9	26.0	27.1
Simulation (EPQ/R-L)	34.4	42.7	22.9
Psychophysiological activation (QPF/R)	43.8	30.2	26.0
Phobias/total score (IP-R)	48.9	43.8	7.3
Calamities (IP-1)	46.9	42.7	10.4
Social phobia (IP-2)	51.0	45.8	3.1
Repellent animals (IP-3)	39.6	43.8	16.6
Departure (IP-4)	45.8	44.8	9.4
Physicians and blood (IP-5)	57.3	33.3	9.4
Depressive symptoms	17.7	57.3	25.0
Obsessive–compulsive (MOCQ/R)	54.1	37.5	8.3
Checking (MOCQ/R-1)	56.3	31.2	12.5
Cleaning (MOCQ/R-2)	53.1	39.6	7.3
Doubting/ruminating (MOCQ/R-3)	77.1	19.8	3.1

Table 5 shows the frequency of the main responses obtained from CBA 2.0 anamnestic Schedule 4.

**Table 5:** Information derived from CBA 2.0 Schedule 4 – answers' frequency distributions.

Variable	Male	Female	Statistic test	*p*
Romantic relationship (%)	48.4	70.7	χ2(1)=4.53^a^	0.03
Employed (%)	70.9	58.4	χ2(1)=1.40^a^	0.23
Problem with justice (%)	12.9	7.7	χ2(1)=0.67^a^	0.41
Smoking habit (%)	22.5	35.4	χ2(1)=1.60^a^	0.20
Overuse of alcohol (%)	0	10.8		0.09^b^
Abnormal appetite (%)	29.0	29.2	χ2(1)=0.00^a^	0.98
Eating habits (%)				
Frequent snacks	58.1	50.8	χ2(1)=0.45^a^	0.50
Short lunch breaks	12.9	10.8	χ2(1)=0.09^a^	0.76
Fast eating	48.4	36.9	χ2(1)=1.14^a^	0.28
Frequent intentions of eating less to reduce weight	58.0	49.2	χ2(1)=0.66^a^	0.42
Eat continuously when tired or nervous	16.1	20.0	χ2(1)=0.21^a^	0.65
Eat lightly when tired or nervous	6.4	4.6	χ2(1)=0.14^a^	0.70
Eat too much and always have a great appetite	16.1	30.8	χ2(1)=2.33^a^	0.13
Unsuccessful attempts to follow a diet	64.5	46.2	χ2(1)=2.83^a^	0.09
Health problems (%)	58.0	69.2	χ2(1)=1.16^a^	0.28
Hypertension	50.0	35.5	χ2(1)=1.12^a^	0.28
Thyroidism	22.2	40.0	χ2(1)=1.79^a^	0.18
High cholesterol level	11.1	2.2	χ2(1)=2.24^a^	0.13
Diabetes	50.0	6.7	χ2(1)=15.6^a^	0.00
Other	22.2	31.1	χ2(1)=0.49^a^	0.48
Physical pain (%)	38.8	62.2	χ2(1)=2.83^a^	0.09
Sleep problems (%)	38.8	35.5	χ2(1)=0.06^a^	0.80
Traumatic Experiences (%)	38.8	57.8	χ2(1)=1.84^a^	0.17
Psychological problems (%)				
Neither	87.1	81.5		0.84
Mild to moderate	12.9	16.9		
Severe to very severe	0	1.5		
Suicide attempt (%)	6.4	1.5	χ2(1)=1.67^a^	0.19
Psychiatric drugs (%)	0	13.8		0.03^b^
Past psychiatric/psychological visits (%)	45.2	43.1	χ2(1)=0.04^a^	0.85
Willingness to start psychological therapy (%)			χ2(3)=8.49	0.04
Yes, even if long	35.5	56.9		
Yes, only if brief	6.4	12.3		
Unsure	22.6	18.5		
No	35.5	12.3		

a The statistic has been calculated on a 2 × 2 table.

b Fisher exact test.

Significant positive correlations were found between eating disorders and eight clinical scales of the MMPI-2. These include Hs (*r = 0*.298, *p = 0*.019), D (*r = 0*.331, *p = 0*.009), Pd (*r = 0*.340, *p = 0*.007), Pt (*r = 0*.596, *p* < 0.001), Pa (*r = 0*.264, *p = 0*.038), Sc (*r = 0*.481, *p* < 0.001), Ma (*r = 0*.266, *p = 0*.037), Si (*r = 0*.464, *p* < 0.001). No association was observed with Hy (*r = 0*.058, *p* < 0.653) and Mf (*r = −0*.124, *p* = 0.338). We also found significant correlations between eating disorders and trait anxiety (*r = 0*.410, *p = 0*.002), Extraversion/Introversion (*r = −0*.336, *p = 0*.008), neuroticism/stability (*r = 0*.346, *p = 0*.006), somatic symptoms (*r = 0*.343, *p = 0*.013), depressive symptoms (*r = 0*.596, *p* < 0.001), and obsessive-compulsive symptoms (*r = 0*.555, *p* < 0.001). Then only variables which showed a significant correlation were considered in the subsequent analysis. The following stepwise regression analysis identified the Pt scale as the most significant predictor of eating disorders, explaining the 35% of the variance (beta = 0.436; *t =* 5.749; *p* < 0.001; *R*^2=^ 0.35). In the second stepwise regression analysis, the first model indicated that obsessive-compulsive symptoms were a significant predictor (β = 0.587; *t =* 4.863; *p* < 0.001; *R*^2=^ 0.34). The analysis indicated that adding depressive symptoms as a predictor resulted in a significant *R*^2^ variation (ΔR^2^ = 0.17; *p* < 0.001). Thus the final model explained 51% of the variance (*R*^2^ = 0.51) with obsessive-compulsive (β = 0.462; *t =* 4.208; *p* < 0.001) and depressive symptoms (β = 0.430; *t* = 3.918; *p* < 0.001) as significant predictors of eating disorders.

**Figure 1:**
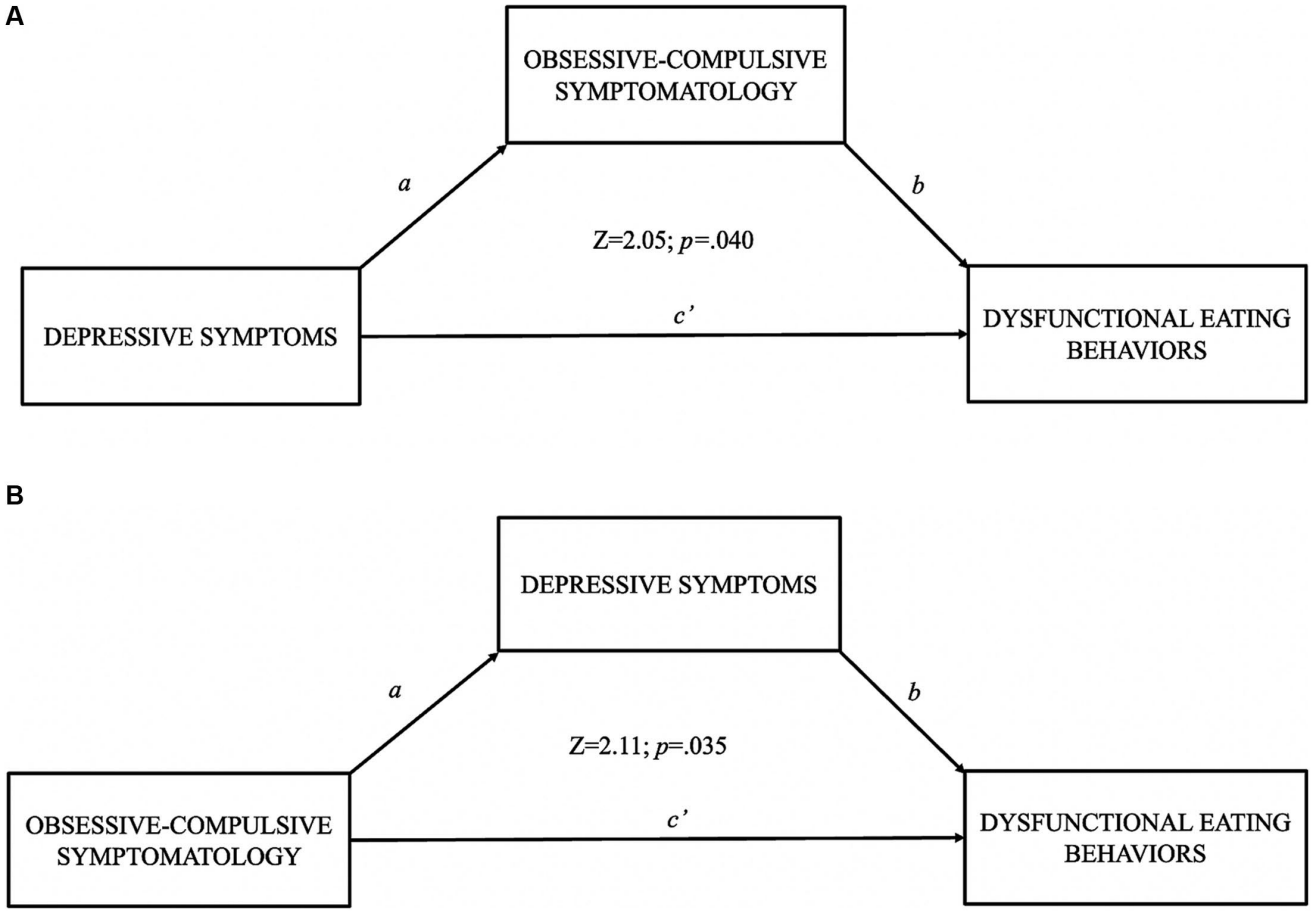
Results of mediation model where **(A)** used obsessive-compulsive symptomatology as mediator between depressive symptoms and dysfunctional eating behaviors; **(B)** used depressive symptoms as mediator between obsessive-compulsive symptomatology and dysfunctional eating behaviors.

We created two mediation models based on regression results. The first model explored the relationship between depressive symptoms (X) and dysfunctional eating behaviors (Y), using obsessive-compulsive symptoms as a mediator. The results showed that the significant indirect relationship between depressive symptoms and dysfunctional eating behaviors was partially and positively mediated by obsessive-compulsive symptoms (*b* = 1.10; SE = 0.54; *Z* = 2.05; *p = 0*.040) and accounting for obsessive-compulsive symptoms still maintained a positive effect due to depressive symptoms (*b* = 4.43; SE = 0.86; *Z* = 5.14; *p* < 0.001). In the second mediation model, the relationship between obsessive-compulsive symptoms (X) and dysfunctional eating behaviors (Y), using depressive symptoms as a mediator, was examined. The results showed that the significant indirect relationship between obsessive-compulsive symptoms and eating behaviors was partially and positively mediated by depressive symptoms (*b* = 1.22; SE = 0.58; *Z* = 2.11; *p = 0*.035), accounting for the mediator still maintained an effect of obsessive-compulsive symptoms on eating disorders (*b* = 3.80; SE = 0.84; *Z* = 4.52; *p < 0*.001). In summary in mediation models, depressive and obsessive-compulsive symptomatology both indicate partial positive mediation toward dysfunctional eating behaviors. Results of mediation analysis are reported in Figure 1.

## 4. Discussion

In the current study, the role of psychopathological factors and personality dimensions on dysfunctional eating behaviors in individuals with obesity was evaluated. The scales were used taking into account the guidelines that suggest which instruments should be applied to assess individuals with obesity who visit a psychologist for treatment counselling (Guerrini Usubini et al., 2021). The results on the scales used are in line with those reported in the literature (Chen et al., 2021). Our sample consists mainly of women (68%), with more than half of our participants belonging to obesity class III and most reporting medical problems including diabetes, thyroidism, high cholesterol and hypertension, confirming the findings of previous studies (Barbuti et al., 2022). Although most participants show MMPI-2 profiles in the normal range, a significant percentage of subjects reported high scores (T ≥ 65) in several clinical scales. Specifically, we found high proportions on the Hs (54.2%), Hy (20.8%), D (19.8%) and Pd (18.8%) scales. It should be noted that a high percentage of subjects fall within the pathological range on the Hs and Hy scales, demonstrating the presence of somatic concerns and hypochondria (Rosik, 2005). This is also confirmed by a high percentage of subjects scoring T ≥ 65 on the Health Concerns (HEA) content subscale (36.5%). Similar patterns are also observed in the CBA 2.0 results. There is a significantly higher percentage of participants in our study (26%) who scored high (>75) on the QPF-R, which is indicative of mild to severe psychophysiological disorders. In addition, 25% of our sample reported depressive symptomatology. As hypothesized, our sample showed more pathological scores on the EDI than those found in normal-weight subjects without ED (Adami et al., 1994), especially on the three subscales assessing weight-related attitudes and behaviors. The literature suggests that comorbidity rates between ED and obesity are high and this association exposes individuals to a higher risk of medical, psychological and social complications (da Luz et al., 2018; Chu et al., 2019). Furthermore, the hypothesis that obesity may be the result of an ED has been increasingly accepted in recent years (Weiss et al., 2020), suggesting that in these cases the diagnosis of obesity could be secondary to a well-known clinical condition (such as BED or bulimia). Individuals with obesity and those with an ED share common psychological and behavioral characteristics (Iorio et al., 2000). According to Prefit et al. (2019), dysfunctional eating behaviors could represent maladaptive strategies for regulating negative emotional states. For example, higher levels of depression may induce dyscontrolled eating as a dysfunctional strategy, resulting in a possible increase of ED and vice versa (Sander et al., 2021). Our results showed that depressive symptoms, obsessive-compulsive symptoms, and psychasthenia play a key role in the perpetration of dysfunctional eating behaviors. Psychasthenia represents the inability to resist specific actions or thoughts, regardless of their maladaptive nature (Brucoli et al., 2019). In our sample, 9.4% of participants report higher scores on the Pt clinical scale, showing difficulties in decision-making, generalized anxiety, distress, somatic symptoms, obsessive ideas, compulsions, and disorganized thinking. Psychasthenia represents a construct that is no longer commonly used today (Brucoli et al., 2019) however it is often considered very close to obsessive-compulsive disorder (Monaco et al., 2005), but also includes symptomatology that is very common to depression, such as low mental energy, fatigability, and difficulties in mental concentration (Deniker and Ganry, 1992). In fact, in our mediation models, depressive and obsessive-compulsive symptomatology both indicate positive mediation toward ED. This means that the combination of depressive and obsessive-compulsive symptomatology may exacerbate or contribute to the occurrence of dysfunctional eating behaviors. Some individuals with obesity have intrusive thoughts about food and eating compulsions on a dysmotivational basis (Weiss et al., 2020). The inability to control such impulses and the lack of cognitive energy needed to adopt functional strategies can lead to a paradoxical increase in the frequency of such thoughts and triggering behavioral impulse. Recent studies have shown that obesity could be related to decision-making deficits in relation to food that would appear to be very similar to those observed in addictive behaviors (Chen et al., 2018; Leigh and Morris, 2018). The lack of inhibition and lack of control appears to be related to an alteration of reward- and gratification-related circuits (Leigh and Morris, 2018). Similarly, several studies demonstrated the association between depression and abnormal activity within dopaminergic regions of the reward system, particularly with regard to food (Milaneschi et al., 2019). In fact, elevated depressive symptomatology appears to be associated with increased emotional eating, particularly in adults with obesity with elevated depressive symptoms (Privitera et al., 2015).

## 5. Conclusion

Careful psychiatric and psychological assessment, as well as an intervention focusing on eating behavior appears to be essential, even before obesity treatment (Packianathan et al., 2002). For this reason, we have attempted to identify some psychopathological features that should be the specific focus of psychological interventions. Psychiatric evaluations are widely recommended during the assessment performed before bariatric surgery because personality variables, psychopathological and psychosocial factors can have a significant impact on short- and long-term postsurgical outcomes (Federico et al., 2019; Barbuti et al., 2022). Our results suggested that difficulties related to impulse and behavioral control, along with negative and dysphoric emotions, may be associated with difficulties in eating behavior. These aspects may compromise the implementation of adequate eating behaviors and lead to the choice to undertake surgery to correct the inappropriate eating behavior, without engaging in appropriate psychological and behavioral interventions focused on impulse control and emotional regulation. Although bariatric surgery shows good efficacy up to 10 years after surgery, many patients regain weight between 6 months and 1 year after surgery (Marek et al., 2017b). The guidelines state what should be assessed in the pre-surgical stage, including psychiatric symptoms and problematic eating behaviors (Forestieri, 2015; Sogg et al., 2016; Miller-Matero et al., 2018). However, there is a lack of explicit reference to the fact that people with psychopathological problems should follow a psychotherapeutic program of good duration and try to lose weight with the support of a psychologist, before accessing surgery. In addition, after surgery, rehabilitation must be undertaken again to prevent weight gain. Promising results in the literature seem to come from modern clinical therapeutic approaches that, alongside traditional cognitive behavioral therapy (CBT), combine personalized procedures for long-term weight loss maintenance (e.g., cognitive behavioral therapy for obesity CBT-OB; Dalle Grave et al., 2020) or the promotion of “psychological flexibility” and self-regulatory skills (e.g., the Acceptance and Commitment Therapy – ACT; Cattivelli et al., 2021).

We are aware that our study has some limitations. Our sample is composed mainly of women and future studies should include more representative samples. One limitation of our study is the relatively small sample size, so the results should be replicated with a larger sample to allow generalization of our findings. Furthermore, a future aim will be to explore these relationships by including samples of individuals with obesity who have a clinical diagnosis of ED in comorbidity. Another limitation of our study is the absence of a control group of overweight and normal-weight patients that would have been useful to highlight other differences between patient groups. We chose to use stepwise regression to favor those variables that had a stronger association at the expense of the others; we therefore decided to use a conservative approach to explore relations between the variables involved. In conclusion, although further evidence is needed, our results provide clinicians with preliminary information on psychological factors to be considered and explored during the assessment of patients with obesity, especially in individuals who want to undergo surgery or who have reported repeated failures in weight loss attempts. In addition, these psychopathological aspects should be the target of specific psychological interventions.

## Data availability statement

The datasets presented in this article are not readily available because they contain confidential information relating to the privacy of the participants. Requests to access the datasets should be directed to the corresponding author.

## Ethics statement

The studies involving humans were approved by International Review Board, University of L’Aquila. The studies were conducted in accordance with the local legislation and institutional requirements. The participants provided their written informed consent to participate in this study.

## Author contributions

MM conceived, designed, supervised the work and data analysis. MA and AG contributed to the analysis and interpretation of the data and the drafting of the main manuscript, with support from LR and VDF. ST contributed to the data analysis. CL, ILD, and GDP collected and organized the data. MV contributed to the critical revision and supervision of the manuscript. All authors contributed to the article and approved the submitted version.

## Conflict of interest

The authors declare that the research was conducted in the absence of any commercial or financial relationships that could be construed as a potential conflict of interest.

## Publisher’s note

All claims expressed in this article are solely those of the authors and do not necessarily represent those of their affiliated organizations, or those of the publisher, the editors and the reviewers. Any product that may be evaluated in this article, or claim that may be made by its manufacturer, is not guaranteed or endorsed by the publisher.
